# Dietary protein intake is associated with body mass index and weight up to 5 y of age in a prospective cohort of twins^1^,^2^

**DOI:** 10.3945/ajcn.115.118612

**Published:** 2015-12-30

**Authors:** Laura Pimpin, Susan Jebb, Laura Johnson, Jane Wardle, Gina L Ambrosini

**Affiliations:** 3 Medical Research Council Human Nutrition Research, Cambridge, United Kingdom;; 4 Nuffield Department of Primary Care Health Sciences, University of Oxford, Oxford, United Kingdom;; 5 Centre for Exercise, Nutrition and Health Sciences, School for Policy Studies, University of Bristol, Bristol, United Kingdom;; 6 Cancer Research UK Health Behaviour Research Centre, Department of Epidemiology and Public Health, University College London, London, United Kingdom; and; 7 School of Population Health, The University of Western Australia, Perth, Western Australia

**Keywords:** protein, growth, BMI, weaning, children, longitudinal studies, macronutrients, obesity

## Abstract

**Background:** Few large epidemiologic studies have investigated the role of postweaning protein intake in excess weight and adiposity of young children, despite children in the United Kingdom consistently consuming protein in excess of their physiologic requirements.

**Objective:** We investigated whether a higher proportion of protein intake from energy beyond weaning is associated with greater weight gain, higher body mass index (BMI), and risk of overweight or obesity in children up to 5 y of age.

**Design:** Participants were 2154 twins from the Gemini cohort. Dietary intake was collected by using a 3-d diet diary when the children had a mean age of 21 mo. Weight and height were collected every 3 mo, from birth to 5 y. Longitudinal models investigated associations of protein intake with BMI, weight, and height, with adjustment for age at diet diary, sex, total energy intake, birth weight/length, and rate of prior growth and clustering within families. Logistic regression investigated protein intake in relation to the odds of overweight or obesity at 3 and 5 y of age.

**Results:** A total of 2154 children had a mean ± SD of 5.7 ± 3.2 weight and height measurements up to 5 y. Total energy from protein was associated with higher BMI (β = 0.043; 95% CI: 0.011, 0.075) and weight (β = 0.052; 95% CI: 0.031, 0.074) but not height (β = 0.088; 95% CI: −0.038, 0.213) between 21 mo and 5 y. Substituting percentage energy from fat or carbohydrate for percentage energy from protein was associated with decreases in BMI and weight. Protein intake was associated with a trend in increased odds of overweight or obesity at 3 y (OR = 1.10; 95% CI 0.99, 1.22, *P* = 0.075), but the effect was not statistically significant at 5 y.

**Conclusion:** A higher proportion of energy from protein during the complementary feeding stage is associated with greater increases in weight and BMI in early childhood in this large cohort of United Kingdom children.

See corresponding editorial on page 303.

## INTRODUCTION

There is substantial evidence from a large, high-quality randomized control trial that the higher protein content of formula milk than breast milk is associated with adverse infant and child outcomes (1, 2). Several meta-analyses of the differential effect of breast compared with formula feeding on subsequent adiposity propose that the differing protein content of these exposures may be one of the main determining factors (3, 4). Although investigations of other dietary macronutrients in relation to childhood obesity have not found strong associations for fat or carbohydrate (5), the role of protein intake on adiposity beyond the weaning period is unclear. The introduction of solid foods in the postweaning phase increases children’s protein intake in comparison with the infant feeding period, whereas protein requirements decrease, along with growth rates (6). Children’s postweaning protein intake may therefore exceed their physiologic requirements, including the proportion of total energy intake provided by protein. The growth hormone/insulin-like factor 1 axis may be stimulated by excess protein intake and drive early differentiation and proliferation of adipocytes (7). Increased protein intake has been identified as an important dietary determinant of circulating insulin-like factor 1 concentrations in humans (8), lending further support to this suggested mechanism. There is evidence from some prospective studies of an association between protein intake in children aged <2 y [both as total protein (g/d) and as the proportion of energy from protein (%Epro)^8^] and measures of size and risk of obesity in childhood and adolescence (7, 9–13), although other similar studies show no association (14–16). One common limitation of these studies is their small sample size, which may not allow detection of small association effects. Outcomes are also usually measured on only 1 or 2 occasions with large intervals between them; although this limits participant burden and allows for longer follow-up periods, it reduces sensitivity for detecting variation in growth in longitudinal analyses. The association between protein intake during infancy and size in later childhood or adolescence does not allow adjustment for growth in the intervening period, which may have a stronger impact on the outcome measure than early protein intake (17), potentially resulting in spurious or even null associations. Despite adjustment for total energy intake in many analyses, the effect of increasing protein in these studies cannot easily be distinguished from the reciprocal decrease in intake of other macronutrients.

Here we use frequently repeated measurements of growth collected in a large birth cohort to investigate the role of protein during the complementary feeding period on child anthropometric measurements. We hypothesized that protein intake, as a proportion of total energy intake, would be prospectively associated with increases in weight, height, and BMI (in kg/m^2^) up to ages 3 and 5 y, as well as increased odds of overweight and obesity at ages 3 and 5 y.

## METHODS

### Study population

The study sample were participants in the Gemini study, a population-based birth cohort of twins recruited by using Office for National Statistics birth registration data for all twins born in England and Wales from March to December 2007 (*n* = 6754). A total of 3435 families (51%) agreed to be contacted, of whom 2402 (70% of those contacted and 39% of all eligible families) returned a baseline questionnaire when the twins had a mean ± SD age of 8 ± 2.2 mo (18). Ethical approval for Gemini was granted by the University College London Committee for the Ethics of Non–National Health Service Human Research, and all aspects of the data collection and storage were in accordance with the standards stipulated by this committee.

### Anthropometric variables

Parents were invited to report weights and heights recorded by health care professionals in the “Red Book,” along with the date of each measurement, when the twins were 8, 15, and 24 mo old. Where professional-recorded weights were unavailable, parents were asked to measure and record their children’s weights (3.6% of data). When the twins were 24 mo old, parents were sent electronic weighing scales (Tanita) and height measurement wall charts along with detailed instructions on how to measure, record, and report their twins’ weights and height every 3 mo. Measurements were recorded on the height chart and reported to researchers up to a median age of 55.0 (IQR: 42.0–60.7) mo for the last recorded measurement at the time of this analysis.

BMI was calculated at each age. At 3 y (36 mo, with data points ranging from 33 to 39 mo) and 5 y (ranging from 57 to 63 mo), each child’s BMI was classified according to the International Obesity Task Force (IOTF) age- and sex-specific cutoffs into thin, normal weight, overweight, or obese, based on adult BMI cutoffs at 18 y (19, 20). If more than one BMI measurement was available within the 6-mo interval around the 36- or 60-mo time points and overweight/obesity status was discordant at the 2 times, the BMI measurement closest to exactly 36 or 60 mo was used to classify overweight/obesity status.

### Dietary variables

Parents (and caregivers when children were not in parents’ care) were requested to record all food and beverage consumption for each twin by using a 3-d estimated diet diary with portions reported by using household measures and age-specific portion size photographs (21) between November 2008 and August 2009, when the twins had a mean ± SD age of 21 ± 1.2 mo (range: 17.3–34.2 mo). A total of 2714 diaries were returned. Diaries that recorded only 1 d (*n* = 122), spanned more than 28 d from first to last recorded day (*n* = 132), were not completed within the 17- to 28-mo age range (*n* = 2), or came from twins of unknown zygosity (*n* = 26) (22) were excluded, providing dietary data for 2432 individuals (50.6% of the cohort).

Diet diaries were coded and linked to British food composition tables at Medical Research Council Human Nutrition Research (Cambridge) (23) to provide mean daily intakes of total energy (kJ), protein (g/d), mean %Epro, mean proportion of total energy from fat (%Efat), and mean proportion of total energy from carbohydrate (%Ecarb). Age at diary entry was calculated by using date of birth and date of first day of diet diary recording.

### Covariates

Parents were asked to report the children’s sex and twin zygosity, ethnicity, household socioeconomic status (SES) (24), and the feeding method used in the first 3 mo of life (coded into 7 categories ranging from exclusively bottle fed to exclusively breastfed). Maternal BMI when the twins had a mean age of 8 mo was categorized as underweight (≤18.49), normal weight (18.5–24.9), overweight (25–29.9), or obese (≥30) (25). Ethnicity was coded as white or other, due to a small number of ethnic minority respondents.

### Statistical analysis

#### Descriptive analysis

Sample demographics, along with other covariates, were described according to quintiles of %Epro. Tests for trends across quintiles were conducted by using the median value in each protein category as a continuous outcome variable in the linear regression models for each descriptive as a predictor variable, respectively (**Table 1**).

**TABLE 1:** Dietary, demographic, and anthropometric variables by quintiles of proportion of energy from protein intake at 21 mo in the Gemini study

	Quintiles of proportion of energy intake from protein^1^										
	1		2		3		4		5	
	*n*	Value	*n*	Value	*n*	Value	*n*	Value	*n*	Value	*P* value^2^
Age at diet diary, mo	401	20.9 ± 1.0^3^	433	20.9 ± 0.9	430	20.9 ± 1.0	449	21.0 ± 1.0	441	21.0 ± 1.1	0.926
Energy from protein, %	401	12.6 ± 1.1	433	14.5 ± 0.4	430	15.8 ± 0.3	449	16.9 ± 0.4	441	18.8 ± 1.3	<0.001
Energy from fat, %	401	34.3 ± 5.5	433	35.1 ± 4.5	430	35.6 ± 4.5	449	36.6 ± 4.4	441	35.9 ± 4.9	<0.001
Energy from carbohydrate, %	401	53.1 ± 5.5	433	50.4 ± 4.5	430	48.7 ± 4.5	449	46.6 ± 4.4	441	45.4 ± 5.0	<0.001
Total intake, g	401	1195.5 ± 279.4	433	1191.8 ± 246.7	430	1244.6 ± 257.4	449	1258.3 ± 275.6	441	1218.4 ± 261.9	0.002
Total energy intake, kJ	401	4433.9 ± 865.1	433	4391.4 ± 737.1	430	4388.7 ± 752.6	449	4351.2 ± 756.7	441	4147.2 ± 732.6	<0.001
Birth weight, kg	394	2.5 ± 0.6	426	2.5 ± 0.5	429	2.5 ± 0.5	440	2.5 ± 0.5	436	2.47 ± 0.5	0.646
Birth length, cm	155	47.9 ± 3.7	148	47.6 ± 3.5	177	47.7 ± 4.0	170	47.5 ± 5.3	188	47.6 ± 4.2	0.858
Slope of BMI up to 21 mo^4^	328	1.1 ± 0.0	364	1.1 ± 0.0	341	1.1 ± 0.04	373	1.1 ± 0.0	379	1.1 ± 0.0	0.122
Slope of weight up to 21 mo^4^	401	1.4 ± 0.1	433	1.4 ± 0.1	430	1.4 ± 0.06	447	1.4 ± 0.1	441	1.4 ± 0.1	0.495
Slope of height up to 21 mo^4^	328	1.1 ± 0.0	364	1.1 ± 0.0	341	1.1 ± 0.03	374	1.1 ± 0.0	379	1.1 ± 0.0	0.949
BMI at 36 mo	190	16.0 ± 1.7	201	16.1 ± 1.3	213	16.1 ± 1.3	226	16.1 ± 1.3	197	16.2 ± 1.4	0.155
BMI at 60 mo	123	15.3 ± 1.4	145	15.5 ± 1.2	103	15.3 ± 1.1	150	15.2 ± 1.2	144	15.4 ± 1.3	0.980
Weight at 36 mo	201	14.3 ± 1.8	207	14.3 ± 1.6	222	14.4 ± 1.6	233	14.2 ± 1.5	208	14.5 ± 1.8	0.249
Weight at 60 mo	126	18.0 ± 2.4	145	18.5 ± 2.1	103	18.6 ± 2.0	154	18.3 ± 2.1	144	18.4 ± 2.4	0.480
Height at 36 mo	190	94.3 ± 3.6	201	94.2 ± 3.9	213	94.4 ± 3.7	226	93.9 ± 3.6	197	94.7 ± 3.6	0.669
Height at 60 mo	123	108.6 ± 5.3	145	109.2 ± 4.2	103	110.0 ± 4.1	150	109.5 ± 5.0	144	109.1 ± 4.7	0.335
Sex, %											
Male	209	52.1	220	50.8	218	50.7	210	46.8	191	43.3	0.058
Female	192	47.9	213	49.2	212	49.3	239	53.2	250	56.7	—
Zygosity, %											
Monozygotic	127	31.7	132	30.5	135	31.4	149	33.2	147	33.2	0.879
Dizygotic	274	68.3	301	69.5	295	68.6	300	66.8	294	66.8	—
Maternal ethnicity, %											
White	363	90.5	395	91.2	380	88.4	403	89.8	385	87.3	0.104
Other	38	9.5	38	8.8	50	11.6	46	10.2	56	12.7	—
Socioeconomic status, %											
High	66	16.5	61	14.1	53	12.3	54	12.1	62	14.1	0.053
Medium	38	9.5	75	17.4	60	14.0	58	13.0	65	14.8	—
Low	296	74.0	296	68.5	317	73.7	335	74.9	312	71.1	—
Missing	1	0.2	1	0.2	0	0	2	0.5	2	0.5	—
Feeding method in first 3 mo, %											
Entirely breastfeeding	75	18.7	71	16.4	59	13.8	87	19.4	75	17.0	0.420
Mostly breast, some bottle	81	20.2	94	21.7	93	21.6	92	20.5	87	19.7	
Equally breast and bottle	43	10.7	40	9.2	46	10.7	46	10.2	38	8.6	—
Mostly bottle, some breast	53	13.2	70	16.2	77	17.9	89	19.8	76	17.2	—
Almost entirely bottle	51	12.7	69	15.9	67	15.6	55	12.3	64	14.5	—
Entirely bottle	84	21.0	75	17.3	77	17.9	65	14.5	80	18.1	—
Other	14	3.5	14	3.2	11	2.6	15	3.4	21	4.8	—
Maternal BMI (in kg/m^2^) at baseline, %											
≤18.49	10	2.5	4	0.9	6	1.4	5	1.1	15	3.4	0.009
18.5–24.9	230	57.4	261	60.3	281	65.4	229	57.7	269	61.0	—
25–29.9	115	28.7	117	27.0	111	25.8	140	31.2	103	23.4	—
≥30	40	10.0	44	10.2	25	5.8	45	10.0	48	10.9	—
Missing	6	1.5	7	1.6	7	1.6	0	0	6	1.4	—

1 Quintile cutoffs for %Epro: 8.3 ≤ quintile 1 < 13.8 ≤ quintile 2 < 15.1 ≤ quintile 3 < 16.3 ≤ quintile 4 < 17.4 ≤ quintile 5 < 25.7.

2 Test for trend by linear regression by using median value for each quintile of energy from protein as a continuous variable. χ^2^ test for trend in categorical variables.

3 Mean ± SD (all such values).

4 Prior growth predicted by using a mixed-effects model regressing repeated anthropometric measures between birth and diet diary on age.

#### Growth models

Linear mixed-effect models were developed by using repeated measurements of BMI, weight, and height from the first measurement available after the diet diary (median age: 24.1 mo; IQR: 22.1–24.8 mo) up to 60 mo of age (and up to 36 mo of age) as the outcome variable, as well as time at measurement (wk) as the level 1 predictor and measures of protein intake as the level 2 predictor variable. The best-fitting models (according to likelihood ratio tests) included a random intercept and slope and unstructured covariance between the random effects at both the twin pair and the individual level.

All adjusted models included rate of prior growth to account for growth between birth and the last measurement before time of diet diary (median age: 15.4 mo; IQR: 12.9–18.2 mo).

#### Modeling protein intake and obesity outcomes

To account for variation in energy intake (EI) by growth and development, we modeled protein intake as the %Epro. The %Epro was modeled as both a continuous variable and a categorical variable (quintiles) to investigate associations between amounts of intake present in the sample population and anthropometric measurements. The regression coefficient in the mixed-effect model of continuous protein intake can therefore be interpreted as the effect on the growth measure resulting from a 1-unit increase in %Epro. Coefficients for quintiles of %Epro as a categorical variable are interpreted as the effect on the growth measures associated with membership of each respective quintile of %Epro intake, relative to the first quintile (the reference category).

It was not possible to accurately describe intakes as grams of protein per kilogram of body weight (g/kg per day), because the weight of the children was not collected at the exact time of the diet diary record.

Nutrient density substitution models were applied to investigate the effect of replacing %Epro with proportion of energy from fat (%Efat) or carbohydrate (%Ecarb) on BMI, weight, and height. The basic nutrient density substitution model included %Efat and %Ecarb and total EI but omitted %Epro. Coefficients for %Efat and %Ecarb were interpreted as the effect of replacing 1% of %Epro with 1% of %Efat or 1% of %Ecarb, respectively, because all other energy sources are held constant by the inclusion of total EI.

#### Adjustment for covariates

Covariates tested for inclusion in the protein intake and growth models (using forward stepwise selection) were sex, age at diet diary entry, zygosity, ethnicity, feeding method in the first 3 mo of life, family SES, maternal BMI at baseline, rate of prior growth, birth weight (or birth length for models of height), and total EI. The final models included only covariates that were statistically significant according to the likelihood ratio test: sex, age at diet diary entry, rate of prior growth, birth weight (or birth length for the height models), and total EI. Height was included only in models where weight was the outcome. An interaction between %Epro and age was tested in the adjusted model to investigate whether relations between %Epro and BMI, weight, or height changed over time.

#### Odds of overweight or obesity at 36 and 60 mo

Logistic mixed-effect models were used to evaluate the effect of protein intake at 21 mo on the odds of obesity or overweight at a mean ± SD age of 36 ± 3 mo and 60 ± 3 mo. Protein intake (%Epro) was modeled as a continuous and categorical variable (quintiles). Nutrient density substitution models including %Efat, %Ecarb, and total EI were also investigated. To adjust for the within-pair clustering, all logistic regression models included a cluster term for family. The same adjustment covariates as in mixed-effect models were included, with size at birth and rate of growth up to diet diary controlled for by including birth weight and predicted rate of weight gain from birth to last measurement before diet diary.

All analyses were conducted with STATA version 12 (StataCorp LP).

## RESULTS

### Demographics and anthropometric variables

Most Gemini diet diary responders provided at least 2 measurements of weight and height after completion of the diet diary and were included in longitudinal models of growth in relation to prior protein intake (*n* = 2154, 89%). Approximately 1000 participants provided data every 3 mo for the remainder of the follow-up period up to 36 mo, decreasing to 655 and 672 participants with measurements of weight and height, respectively, at 60 ± 3 mo. A total of 165 (12%) of the 1385 with BMI data at ∼36 ± 3 mo were classified as overweight or obese, whereas only 65 (6%) of 1058 with BMI data at 60 ± 3 mo were categorized as such, suggesting a potentially greater dropout rate of heavier children. Children who had growth data at 36 mo but not at 60 mo did not differ in their dietary intakes, birth weight, sex, or zygosity from those who had data at 60 mo of age. However, they were less likely to be of white ethnicity, have a higher SES, or be exclusively or almost exclusively breastfed up to 3 mo, and they were slightly younger at diet diary entry (data not shown).

At least 2 measurements of weight and height from birth to age at diet diary were available for 2424 [mean number of 5 (range: 1–8) measurements] and 1932 [mean number of 2 (range: 1–7) measurements] participants, respectively, which allowed modeling of rate of prior BMI, weight, and height growth for inclusion as a potential confounder in adjusted models.

All dietary variables showed significant trends across increasing quintiles of %Epro (Table 1), with greater %Efat and lower %Ecarb in the higher quintiles of %Epro. A greater %Epro was associated with greater total intake of food and beverages (g/d) but lower total EI (kJ), indicative of a lower energy density of the diet. Children in the higher quintiles of %Epro did not have significantly higher mean BMI, weight, or height up to 36 and 60 mo (Table 1). There were was a trend for more girls and medium to high SES families in the higher quintiles of %Epro. No differences were observed across quintiles of %Epro at 21 mo in age at diet diary completion, birth weight or length, prior growth rate, ethnicity, zygosity, or infant feeding method.

### Growth up to 36 and 60 mo

A total of 2154 respondents had a mean ± SD of 6 ± 3 (range: 1–14) measurements of height, weight, and BMI between 21 and 60 mo. Analyses extending follow-up to 60 mo revealed similar associations as those seen up to 36 mo but with a slightly attenuated effect size for BMI (**Table 2**). A 1% greater %Epro at 21 mo was associated with a 0.04 (95% CI: 0.01, 0.07) greater BMI and a 52-g (95% CI: 31, 74 g) greater weight on average at any time point between 21 and 60 mo in adjusted models (Table 2). These associations did not vary over time up to 60 mo (*P*-interaction = 0.061 for BMI and *P*-interaction = 0.271 for weight).

**TABLE 2:** Association between percentage energy from protein at 21 mo and repeated measures of BMI and weight up to 36 and 60 mo by using mixed-effect models in the Gemini study^1^

	BMI (kg/m^2^) 21–36 mo: model 1 (*n* = 2052) and model 2 (*n* = 1697)		Weight (kg) 21–36 mo: model 1 (*n* = 2052) and model 2 (*n* = 2025)		BMI (kg/m^2^) 21–60 mo: model 1 (*n* = 2154) and model 2 (*n* = 1769)		Weight (kg) 21–60 mo: model 1 (*n* = 2154) and model 2 (*n* = 2050)	
	β (95% CI)	*P* value	β (95% CI)	*P* value	β (95% CI)	*P* value	β (95% CI)	*P* value
%Epro								
Model 1 (basic growth model)	0.060 (0.024, 0.094)	0.001	0.043 (0.016, 0.070)	0.002	0.038 (0.007, 0.068)	0.016	0.045 (0.017, 0.073)	0.002
Model 2 (adjusted)	0.062 (0.025, 0.098)	0.001	0.050 (0.029, 0.072)	<0.001	0.043 (0.011, 0.075)	0.009	0.052 (0.031, 0.074)	<0.001
Nutrient density substitution model^2^								
Model 1 (basic growth model)								
%Efat	−0.067 (−0.108, −0.026)	0.001	−0.058 (−0.090, −0.026)	<0.001	−0.048 (−0.084, −0.012)	0.009	−0.063 (−0.096, −0.030)	<0.001
%Ecarb	−0.071 (−0.106, −0.035)	<0.001	−0.059 (−0.086, −0.032)	<0.001	−0.048 (−0.079, −0.017)	0.002	−0.063 (−0.091, −0.034)	<0.001
Model 2 (adjusted)								
%Efat	−0.050 (−0.094, −0.005)	0.028	−0.045 (−0.070, −0.019)	0.001	−0.035 (−0.073, 0.004)	0.076	−0.047 (−0.073, −0.021)	<0.001
%Ecarb	−0.060 (−0.097, −0.021)	0.002	−0.050 (−0.072, −0.028)	<0.001	−0.040 (−0.0730, −0.007)	0.016	−0.052 (−0.074, −0.030)	<0.001

1 All weight models adjusted for height (cm). Model 1 includes macronutrient intake and total EI (kJ). Model 2 includes all variables from model 1 and adjusts for sex, age at diet diary reporting (mo), birth weight (kg), and modeled slope of previous BMI/weight (depending on the outcome anthropometric variable modeled) from birth to time of diary data. EI, energy intake; %Ecarb, proportion of energy from carbohydrate; %Efat, proportion of energy from fat; %Epro, proportion of energy from protein.

2 Basic nutrient density substitution model includes total EI (kJ); coefficients for %Efat and %Ecarb are interpreted as the estimated increase in BMI or weight at the respective time points associated with replacing 1% of %Epro with 1% of %Efat or 1% of %Ecarb, respectively.

Substitution of %Epro for %Ecarb was associated with overall decreased BMI (*P* = 0.016) and weight (*P* < 0.001) between 21 and 60 mo (Table 2). Substitution of %Epro with %Efat, however, was associated with lower weight (β = −0.05; 95% CI: −0.07, −0.02; *P* < 0.001) but only a tendency for a lower BMI (*P* = 0.076) up to 60 mo.

**FIGURE 1:**
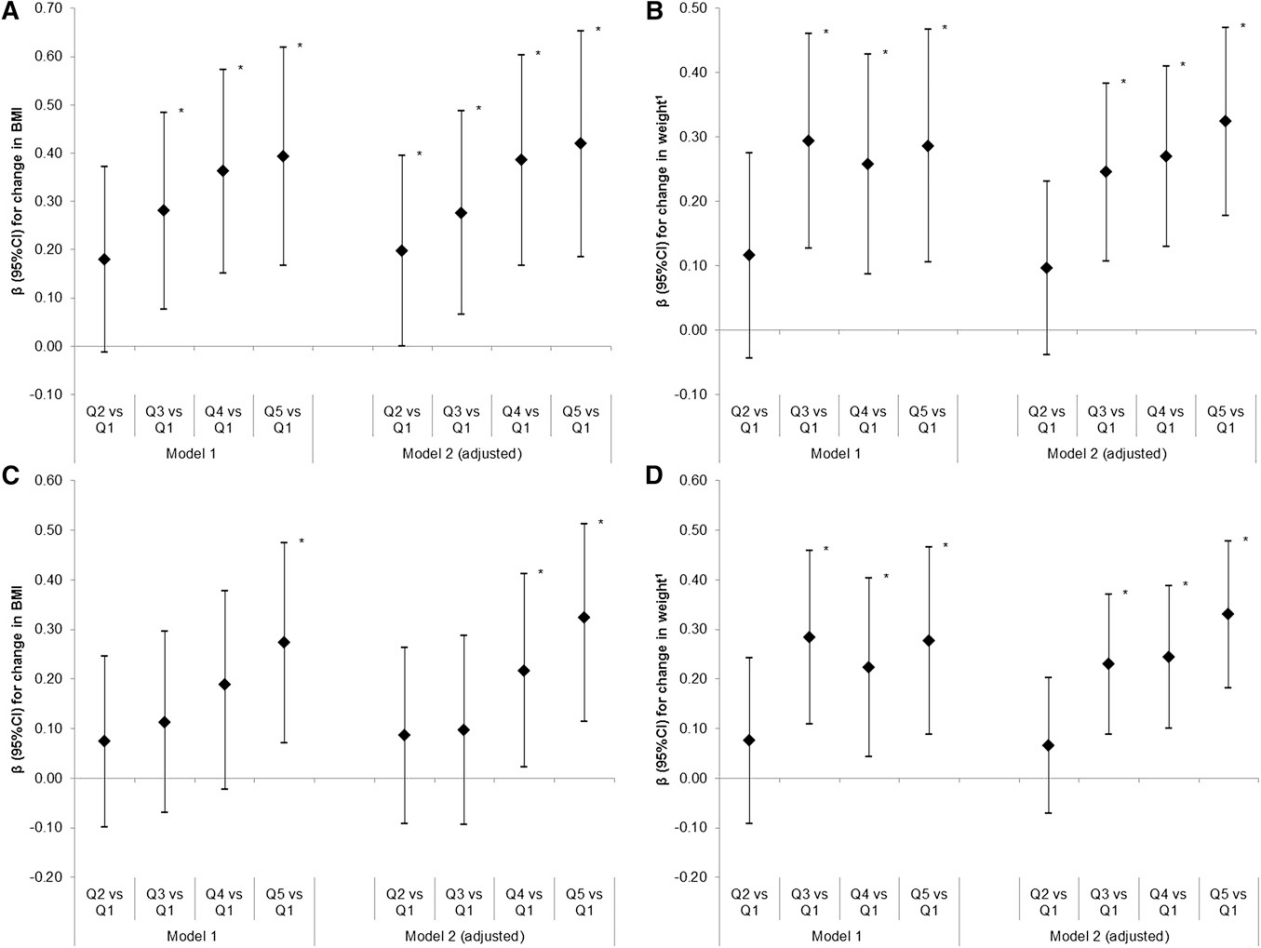
Association between quintiles of percentage energy from protein at 21 mo and repeated measures of BMI (A) and weight (B) up to 36 mo and BMI (C) and weight (D) up to 60 mo in the Gemini study (β as diamond, 95% CI as interval lines). Model 1A and 1B: *n* = 2052; model 2A: *n* = 1697; model 2B: *n* = 2025; model 1C and 1D: *n* = 2154; model 2C: *n* = 1769; model 2D: *n* = 2050. Model 1 includes quintile of %Epro: 8.3 ≤ Q1< 13.8 ≤ Q2 < 15.1 ≤ Q3 < 16.3 ≤ Q4 < 17.4 ≤ Q5 < 25.7 %Epro and total EI (kJ). Includes total EI (kJ). Model 2 includes all variables from Model 1 and adjusts for sex, age at diet diary reporting (mo), total EI (kJ), birth weight (kg), and modeled slope of previous BMI/weight (depending on the outcome anthropometric variable modeled) from birth to time of diary data. ^1^Weight models also adjust for height (cm). *Estimates significant at *P* < 0.05. EI, energy intake; %Epro, proportion of energy from protein; Q, quintile.

Intake of %Epro ≥16.3% (in the top 2 quintiles) was positively associated with higher BMI [β = 0.217 (95% CI: 0.022, 0.412) and β = 0.323 (95% CI: 0.115, 0.531), respectively] and weight [β = 0.244 (95% CI: 0.101, 0.387) kg and β = 0.330 (95% CI: 0.182, 0.478), kg respectively] between 21 and 60 mo compared with the lowest quintile (8.3–13.7%) in the adjusted models (**Figure 1**A and B). No evidence of an association between %Epro at 21 mo and height between 21 and 36 mo (β = 0.049; 95% CI: −0.080, 0.178; *P* = 0.456) or 60 mo (β = 0.088; 95% CI: −0.038, 0.213; *P* = 0.169) was detected (see **Supplemental Table 1** and **Supplemental Figure 1**).

### Odds of overweight or obesity at 36 and 60 mo

**FIGURE 2:**
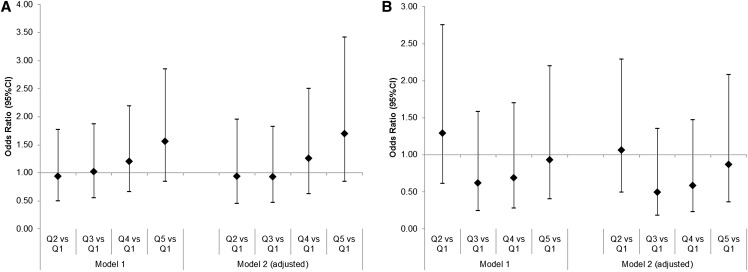
OR of overweight/obesity at a mean ± SD age of 36 ± 3 mo (A) and 60 ± 3 mo (B) by quintiles of mean daily %Epro (OR as diamond, 95% CI as interval lines). Model 1A: *n* = 1385; model 1B: *n* = 1058; model 2A: *n* = 1159; model 2B: *n* = 885. Model 1 includes quintile of %Epro: 8.3 ≤ Q1< 13.8 ≤ Q2< 15.1 ≤ Q3< 16.3 ≤ Q4 < 17.4 ≤ Q5 < 25.7 and total EI (kJ). Model 2 includes all variables from model 1 and adjusts for sex, age at diet diary reporting (mo), birth weight (kg), and modeled slope of previous BMI growth from birth to time of diary data. EI, energy intake; %Epro, proportion of energy from protein; Q, quintile.

There was a trend toward an association between %Epro at 21 mo and the odds of overweight or obesity at 36 mo (OR = 1.10; 95% CI: 0.99, 1.22). Conversely, lower odds of overweight or obesity were observed when %Epro was substituted with %Ecarb (OR = 0.91; 95% CI: 0.81, 1.01; *P* = 0.085) but not with %Efat (OR = 0.92; 95% CI: 0.80, 1.04; *P* = 0.163) in **Table 3**. Although **Figure 2**A suggested a trend toward increased odds of overweight or obesity at 36 mo for the top 2 quintiles of %Epro, they were not statistically significant, (*P* = 0.521 for quintile 4 compared with quintile 1 and *P* = 0.135 for quintile 5 compared with quintile 1). There was no evidence that the odds of overweight or obesity at 60 mo were associated with %Epro at 21 mo in any of the models (Table 3 and Figure 2B).

**TABLE 3:** Association between percentage energy from macronutrients and odds of overweight and obesity at a mean ± SD age of 36 ± 3 mo and 60 ± 3 mo in the Gemini study^1^

	Overweight or obese at 36 ± 3 mo: model 1 (*n* = 1385) and model 2 (*n* = 1159)		Overweight or obese at 60 ± 3 mo: model 1 (*n* = 1058) and model 2 (*n* = 885)	
	OR (95% CI)	*P* value	OR (95% CI)	*P* value
%Epro				
Model 1 (basic model)	1.08 (0.99, 1.18)	0.102	0.94 (0.83, 1.06)	0.313
Model 2 (adjusted)	1.10 (0.99, 1.22)	0.075	0.93 (0.81, 1.07)	0.329
Nutrient density substitution model^2^				
Model 1 (basic growth model)				
%Efat	0.90 (0.81, 1.01)	0.071	1.09 (0.93, 1.26)	0.287
%Ecarb	0.90 (0.82, 1.00)	0.040	1.06 (0.94, 1.21)	0.322
Model 2 (adjusted)				
%Efat	0.92 (0.80, 1.04)	0.163	1.10 (0.91, 1.32)	0.347
%Ecarb	0.91 (0.81, 1.01)	0.085	1.08 (0.93, 1.24)	0.302

1 Model 1 includes macronutrient intake and total EI (kJ) in a logistic regression model with overweight or obesity at a mean ± SD age of 36 ± 3 mo or 60 ± 3 mo as the outcome. Model 2 includes all variables from model 1 and adjusts for sex, age at diet diary reporting (mo), birth weight (kg), and modeled slope of previous BMI growth from birth to time of diary data. EI, energy intake; %Ecarb, proportion of energy from carbohydrate; %Efat, proportion of energy from fat; %Epro, proportion of energy from protein.

2 Basic nutrient density substitution model includes macronutrient intakes and total EI (kJ); coefficients for %Efat and %Ecarb are interpreted as the estimated increase in odds of overweight or obesity at the respective time points associated with replacing 1% of %Epro with 1% of %Efat or 1% of %Ecarb, respectively.

## DISCUSSION

This analysis of longitudinal growth data from >2000 children in the Gemini twin cohort has demonstrated that higher protein intake at 21 mo is associated with higher weight gain and higher BMI (but not height) between 21 and 36 mo and 21 and 60 mo, with no evidence of diminution over time.

There was a trend toward an association between higher protein intake at 21 mo and risk of overweight or obesity up to 36 mo, but this was not statistically significant after all adjustments. However, the use of a single measure of overweight or obesity at one time point, the low prevalence of overweight and obesity in this study population, and the potential for bias through dropout of children of lower SES, varying ethnicity, and children more likely to have been formula fed may have contributed to this weak association. Furthermore, use of IOTF cutoffs, which extrapolate adult BMI cutoffs for normal weight, overweight, and obesity, may not accurately reflect body composition in very young children. Our linear growth models clearly showed a positive association between %Epro and greater weight and BMI of a magnitude that is not negligible. Increased growth in the early years may also be a risk factor for obesity and obesity-related diseases in later life (26).

The positive associations observed of %Epro with weight and BMI but not height in this study match most other prospective observational studies that have investigated the link between protein intake in the first 2 y of life and measures of weight and adiposity but not linear growth into later childhood (7, 9, 11, 13, 17, 27). The largest of these studies followed up to 362 Australian children aged 18 mo to 8 y (17) and found that protein intake at 18 mo, estimated by using a 3-d diet diary, was associated with a higher BMI at 8 y. However, others have reported equivocal results or sex differences: one observed a positive association between BMI *z* score at 4 y and absolute protein intake (g/d) at 17–18 mo (*P* < 0.009) but not with %Epro (28); others, including one cohort study that used longitudinal measurements of BMI from infancy to 15 y (15), found no association with BMI. Two studies found associations between protein intake and BMI in girls but not boys, although one also detected a trend toward an earlier adiposity rebound in the highest %Epro tertile in boys but not girls (10, 29). These were small studies, with only 90–313 participants, so conclusions about the effects of sex or confounders are limited. The mean reported protein intakes in most of these studies were lower (range: 13–15%) than in studies reporting a significant positive association (range: 14–20%). It is also notable that no studies have reported a negative association between a high protein intake and growth in young children.

We observed greater increases in BMI and weight up to 36 mo among children who consumed >15% of their energy from protein, as well as greater increases in BMI and weight up to 60 mo among children who consumed >16.2% of their energy from protein. This supports the nutrition statement from the European Society for Pediatric Gastroenterology, Hepatology, and Nutrition Committee that a >4-g/kg per day protein intake between 18 and 24 mo (equivalent to ∼16% of %Epro) may be associated with increased obesity risk (30). The WHO recommends an amount of intake that meets the requirements of practically all individuals (mean requirement + 2 SD) of 1.03 g/kg per day and 0.97g/kg per day for children aged 18 and 24 mo, respectively (31). When applied to the mean ± SD weight of the Gemini population at these ages (11.0 ± 1.4 kg and 12.3 ± 1.5 kg at 18 and 24 mo, respectively), these translate into recommended intakes ranging from 8.5 to 14.7 g/d (34–59 kcal/d). Given the mean ± SD total energy intake of 1070 ± 235 kcal/d at 18 mo and 1086 ± 209 kcal/d at 24 mo in our sample, the WHO recommendations approximate to a daily protein intake ranging from 3% to 5% of %Epro at this age. Participants in the Gemini study were therefore consuming, on average, 3–5 times the amount of protein intake recommended by WHO. Our nutrient density models showed that replacing energy from protein with energy from fat or carbohydrate was associated with decreases in weight and BMI, suggesting a true growth-promoting effect of protein rather than an association detected due to the correlation between protein, fat, and carbohydrate in the diet. To provide more practically applicable recommendations than upper limits of protein intake to clinicians, parents, and caregivers, further research is required into the specific dietary sources of protein and corresponding dietary patterns, which are more strongly associated with increases in weight and adiposity.

The current study has several strengths, particularly the quality and number of growth measurements. This sample size was much larger than existing studies of protein intake, and associations with measures of body size and growth were examined in both early and mid-childhood. Although twins have lower birth weights than singletons (32), this twin study uses dietary and anthropometric data at ages beyond the period of catch-up growth seen in twins, allowing for greater generalizability to the general population. The prospective analyses adjusted for birth weight, rate of growth before the dietary assessment, and other factors that may influence growth trajectories (4). The estimated 3-d diet diary method is the same as that used in the National Diet and Nutrition Survey carried out in adults and children from 1.5 y of age in the United Kingdom (33) and has been validated for the assessment of intake in children up to 24 mo (34).

An important limitation of this study is the parental reporting of both outcome and exposure variables. Misreporting of food consumption is a potential issue for all dietary assessment methods, although it is not known to what extent parents underreport the dietary intakes of very young children. The potential for underestimating food wastage, resulting in overreporting of actual consumption, is a particular risk in this age group (35, 36). Analysis of the 2007 Australian National Children's Nutrition and Physical Activity Survey found more parents over- than underreporting the intakes of their 2-y-old children (37). However, detailed instructions on how to complete the diet diaries were provided to parents/carers in this study, along with guidelines and age-specific portion size photographs to improve estimation accuracy.

Every effort was made to minimize error in parents’ measurements of child weight and height by providing growth charts and weighing scales and detailed instructions on how to use them. Although differences in the quality of weight and length data between the health professionals’ and parental measurements are possible, the correlation structure between individuals applied in the statistical models accounts for these dependencies in the data. It is also possible that bias was introduced in the anthropometric data through increased dropout over the follow-up period; however, we observed minor differences between those who provided anthropometric measurements at 36 mo but not at 60 mo. Another limitation was the availability of weight and height data only, with no other more precise measures of body composition, such as percentage body fat or skinfold measurements, which may provide stronger evidence of an association between dietary protein and adiposity.

In conclusion, in this United Kingdom cohort of young children, protein intakes >15% of total energy intake were associated with greater weight gain up to 60 mo of age. No association with height was observed, suggesting that high protein intake may be linked to adiposity or lean mass rather than linear growth at these ages. These results provide strong evidence that high protein intake in the first 2 y of life is a risk factor for subsequent childhood weight gain.

## Footnotes

8 Abbreviations used: EI, energy intake; SES, socioeconomic status; %Ecarb, proportion of energy from carbohydrate; %Efat, proportion of energy from fat; %Epro, proportion of energy from protein.
